# Protein Phosphatase Sit4 Affects Lipid Droplet Synthesis and Soraphen A Resistance Independent of Its Role in Regulating Elongator Dependent tRNA Modification

**DOI:** 10.3390/biom8030049

**Published:** 2018-07-11

**Authors:** Bruno Leonardo Bozaquel-Morais, Leonie Vogt, Valentina D’Angelo, Raffael Schaffrath, Roland Klassen, Mónica Montero-Lomelí

**Affiliations:** 1 Instituto de Bioquímica Médica Leopoldo de Meis, Universidade Federal do Rio de Janeiro, Rio de Janeiro 21941-902, Brazil; bozaquel@bioqmed.ufrj.br; 2 Institut für Biologie, Fachgebiet Mikrobiologie, Universität Kassel, 34132 Kassel, Germany; vogt_leonie@outlook.de (L.V.); Vali-DAngelo@gmx.de (V.D.); schaffrath@uni-kassel.de (R.S.)

**Keywords:** soraphen A, Sit4, tRNA modification, Elongator complex

## Abstract

The protein phosphatase Sit4 has been shown to be required for lipogenesis and resistance against the acetyl-CoA carboxylase inhibitor soraphen A. Since Sit4 is also required for biosynthesis of Elongator dependent tRNA modifications such as 5-methoxycarbonylmethyluridine (mcm^5^U), we investigated the relevance of tRNA modifications in lipogenesis and soraphen A response. While *sit4* and Elongator (*elp3*) mutants copy defects in mcm^5^U formation and stress sensitivity, they do not share soraphen A sensitivity and low lipid droplet (LD) phenotypes. In contrast to *sit4*, we found *elp3* mutants to display partial soraphen A resistance and a high LD phenotype. Screening a collection of tRNA modification mutants additionally identified the tRNA pseudo-uridine synthase gene *DEG1* to be required for soraphen A sensitivity. Since *deg1* and *elp3* share high LD and soraphen A resistance phenotypes, these are likely caused by translational defects. In support of this notion, we observe overexpression of tRNA^Gln^UUG suppresses lipolysis defects of *deg1* mutants. Hence, the *sit4* mutation results in a composite defect including tRNA modification deficiency and loss of Snf1 kinase dephosphorylation, which induce opposite effects on LD regulation. Importantly, however, the Snf1 kinase regulatory defects of the phosphatase mutant dominate over effects on LD regulation imposed by loss of the tRNA modification alone.

## 1. Introduction

A key step in lipogenesis is catalyzed by acetyl-CoA carboxylase, which in budding yeast *Saccharomyces cerevisiae* is encoded by the essential *ACC1* gene [1]. Acc1 catalyzes the conversion of acetyl-CoA to malonyl-CoA and its activity is inhibited via phosphorylation by adenosine monophosphate (AMP)-activated protein kinase (AMPK) Snf1 [2,3]. Snf1 kinase activity is regulated by glucose availability and the nutrient sensing pathway (target of rapamycin, TORC1) [2,4,5]. The latter is thought to involve the Sit4-Sap190 protein phosphatase, which antagonizes Snf1 kinase activation. Consistent with this, absence of either Sit4 or Sap190 results in a constitutively phosphorylated and hyperactive Snf1 kinase which results in downregulation of Acc1 activity and a low lipid droplet phenotype, while a *SNF1* gene deletion causes an opposite outcome [2]. The low lipid droplet phenotype of *sit4* mutants was detected using boron-dipyrromethene (Bodipy) fluorescence measurements of stationary phase cultures [2]. In further support of this mutual relationship, cells lacking Snf1 activity are less sensitive towards soraphen A than wild-type yeast, and both *sit4* and *sap190* mutants display strong sensitivity towards the Acc1 inhibitor drug [2].

In addition to its role in regulating lipogenesis, the protein phosphatase complexes Sit4-Sap190 and/or Sit4-Sap185 are also required to maintain Elongator activity [6,7,8,9]. In yeast, the primary function of the Elongator complex, which is composed of subunits Elp1–Elp6 [10,11], lies with formation of the tRNA anticodon modifications 5-methoxycarbonylmethyl-2-thiouridine (mcm^5^s^2^U), 5-methoxycarbonylmethyluridine (mcm^5^U) and 5-carbamoylmethyluridine (ncm^5^U) [12,13]. Mcm^5^s^2^U synthesis additionally requires the presence of a functional sulfur transfer system consisting of Nfs1, Tum1, Urm1, Uba4, Ncs2 and Ncs6 [9]. Absence of Elongator, either alone or in combination with the sulfur transfer system, induces pleiotropic phenotypes including sensitivity against the target of rapamycin (TOR) inhibiting drugs rapamycin and caffeine, cell cycle delays, signaling defects and morphological abnormalities [12,14,15,16,17,18,19]. Most of these composite traits can be rescued by elevating the cellular abundance of tRNAs (i.e., tRNA^Gln^UUG, tRNA^Glu^UUC and tRNA^Lys^UUU) naturally carrying the anticodon modification mcm^5^s^2^U [12,15,17]. Suppression of phenotypes by higher-than-normal levels of tRNAs that critically depend on the anticodon modifications is thought to restore near-normal translation by compensating for ribosomal A-site binding defects [20,21]. While *sit4* and *elp1–elp6* mutants display identical tRNA modification defects [13,22], it remained unknown whether the tRNA modification defect of *sit4* mutants is related to lipid specific phenotypes. Here we compared mutants defective in both, Snf1 regulation and Elongator function (Sit4) with those defective in Elongator alone in relation to general growth and lipid metabolism phenotypes such as soraphen A sensitivity and lipid droplet (LD) content. We provide evidence that loss of the tRNA modification alone in Elongator mutants exerts opposite effects on lipid metabolism as compared to the defect in *sit4*. Further, we demonstrate that not only mcm^5^s^2^U defects but also deficiency in pseudo-uridine formation at tRNA position 38/39 in a *deg1* mutant [23] are associated with a high lipid droplet phenotype that results from delayed lipolysis and confer partial resistance towards Acc1 inhibition by soraphen A.

## 2. Materials and Methods

### 2.1. Strains

Mutants defective in individual subunits of the Elongator complex were obtained from the *S. cerevisiae* deletion collection (OpenBiosystems, Waltham, MA, USA) . Strains were transformed by the lithium acetate method [24]. The plasmids used were pRS425; pKQE (tRNA^Gln^UUG, tRNA^Lys^UUU and tRNA^Glu^UUC genes cloned in pRS425); pRS423 and tQ (tRNA^Gln^UUG gene cloned in pRS423) [17,25,26].

### 2.2. Reagents

Soraphen A was a kind gift from Professor Rolf Müller (Helmoltz-Zentrum für Infektionsforschung, Saarbrücken, Germany) to M.M.L. and R.S. and was kept as a 0.1 mg/mL stock solution in 10% methanol. A crude preparation of zymocin was obtained from culture filtrates of the *Kluyveromyces lactis* killer strain and applied to yeast extract peptone dextrose (YPD) plates as described earlier [27,28]. All other reagents used were from Sigma-Aldrich (St. Louis, MO, USA).

### 2.3. Growth Conditions and Lipid Droplets Quantification 

Yeast cells were grown to stationary phase for 48 h in liquid rich medium (YPD; 1% yeast extract, 2% peptone and 2% glucose) at 30 °C to achieve a high LD content. For LD dynamics, the stationary cells were seeded at low density (optical density (O.D) 600 nm = 0.25) in YPD. At the indicated times cells were fixed in 3.7% formaldehyde and the LD was measured by the liquid fluorescence recovery assay [2,29]. In this assay formaldehyde-fixed cells are added to a medium containing a quenched solution of the hydrophobic fluorescent probe Bodipy containing 5 µM Bodipy 493/503 (Invitrogen, Waltham, MA, USA) and 0.5 M of the fluorescence quencher potassium iodide (KI). After cell addition Bodipy 493/503 enters readily into the cells and fluorescence is measured with a Spectramax M5 fluorimeter (Molecular Devices, San Jose, CA, USA) at 495/510 nm. Fluorescence is normalized to O.D 600 nm and reported as lipid droplet index (LD index).

### 2.4. Drug Assays

*S. cerevisiae* strains were grown for 24 h to stationary phase in YPD or synthetic defined (SD) medium plus appropriate auxotrophic markers, and serial dilutions of the cultures were prepared in sterile-distilled water to 10^7^, 10^6^ and 10^5^ cells/mL. Dilutions were spotted using a replica plater in solid medium in the presence of the drugs indicated. The microtiter based liquid growth assay was done as described [27].

## 3. Results

### 3.1. Analysis of Shared and Non-Shared Phenotypes of sit4 and elp3 Mutants

Mutants defective in individual subunits of the Elongator complex are known to exhibit pleiotropic phenotypes, which can routinely be suppressed by overexpression of the three tRNAs normally carrying the wobble uridine (U_34_) mcm^5^s^2^U modification. Since *sit4* mutants also lack the ability to form modified nucleosides such as mcm^5^s^2^U, mcm^5^U and ncm^5^U [22], we first compared phenotypes of *elp3* and *sit4* mutants and tested their response to overexpression of the three tRNAs. We reasoned that phenotypes caused by mcm^5^s^2^U deficiency but not those resulting from additional impacts of the *sit4* mutation on Snf1 regulation should respond to upregulated tRNA gene dosage.

**Figure 1:**
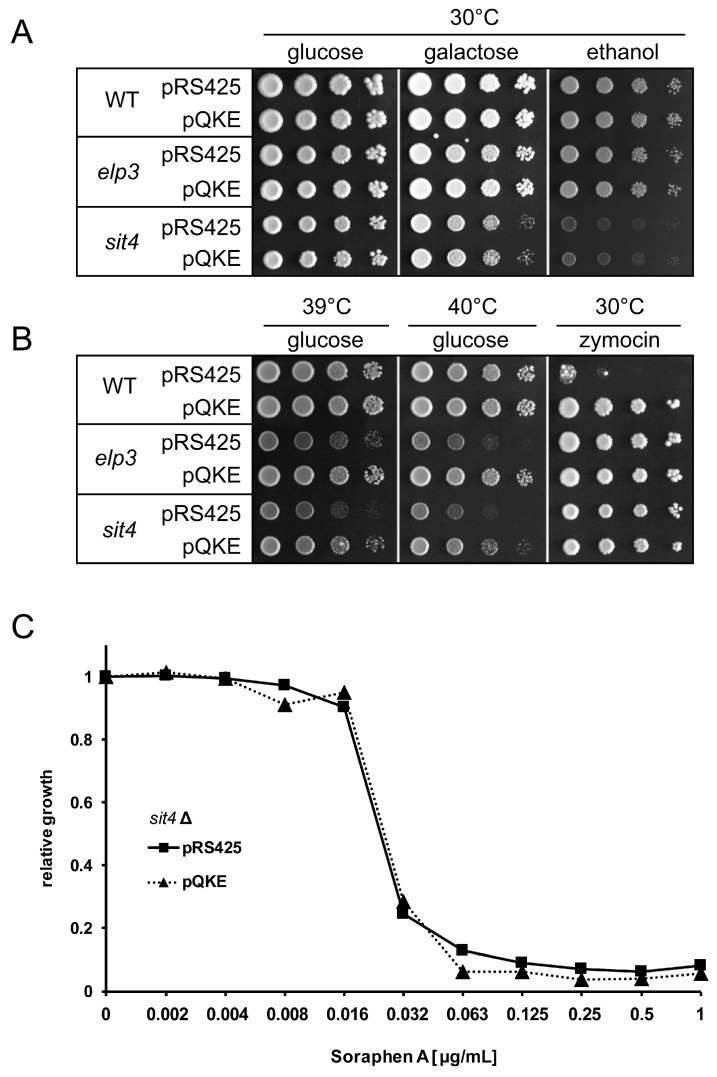
Phenotypes of *elp3* and *sit4* mutants and their responses to overexpression of tRNA^Gln^UUG, tRNA^Lys^UUU and tRNA^Glu^UUC. (**A**) BY4741 wild type (WT), *elp3* or *sit4* mutants were each transformed with empty plasmid pRS425 or pQKE (over-expression of tRNA^Gln^UUG, tRNA^Lys^UUU and tRNA^Glu^UUC) and subjected to drop dilution spot assays involving yeast extract peptone (YP) based media with either glucose, galactose or ethanol as the carbon source. All plates were incubated at 30 °C for 3 days. (**B**) The same strains as in (**A**) were subjected to drop dilution spot assays on yeast extract peptone dextrose (YPD) medium (carbon source glucose) cultivated at 39 °C or 40 °C or on YPD medium supplemented with a crude zymocin preparation. (**C**) Microtiter based measurement of growth inhibition by soraphen A. Relative growth refers to optical density (O.D 600 nm) of microcultures with indicated concentrations of soraphen A as compared to soraphen A free cultures. Measurements were carried out in triplicate and presented as mean with standard deviation.

We transformed *sit4* and *elp3* mutant strains and the isogenic wild type BY4741 with either pRS425 or pQKE. The latter was previously shown to result in overexpression of tRNA^Gln^UUG, tRNA^Lys^UUU and tRNA^Glu^UUC and to suppress most phenotypes of U_34_ modification defects, including temperature sensitivity [12,17]. In addition, overexpression of these three tRNAs or tRNA^Glu^UUC alone results in resistance against the tRNA cleaving toxin zymocin [26,30]. As expected, the wild type carrying the pQKE construct displayed zymocin resistance, whereas the empty vector carrying strain was inhibited (Figure 1).

Since the mcm^5^U deficiency results in non-cleavage of tRNA by zymocin [26,31], *elp3* and *sit4* mutants displayed resistance against the toxin regardless of whether empty vector or pQKE was present, thereby confirming the shared modification defect of the two mutants. In addition, *sit4* and *elp3* mutants displayed comparable growth defects at elevated temperature (39 °C and 40 °C) which can be rescued by overexpression of the three tRNAs. In contrast to this, *elp3* and *sit4* mutants display divergent phenotypes on media with altered carbon sources, which may result from deregulation of Snf1 activity. While *sit4* mutants show growth defects on galactose and ethanol media, the *elp3* mutant did not (Figure 1A,B). Consistently, these *sit4* specific phenotypes are not suppressed by tRNA overexpression, suggesting they are caused by loss of additional Sit4 functions not necessarily linked to the tRNA modification defects. We further investigated whether overexpression of the three tRNAs that rescued temperature sensitive growth of the *sit4* mutant also suppressed the previously observed strong soraphen A sensitivity of this mutant [2]. Using a sensitive microtiter based liquid growth assay over a broad range of soraphen A concentrations, we found no difference in the soraphen A sensitivity between pRS425 and pQKE carrying *sit4* strains (Figure 1C).

### 3.2. Role of 5-Methoxycarbonylmethyluridine Modification in Soraphen A Resistance and Lipid Droplet Dynamics

**Figure 2:**
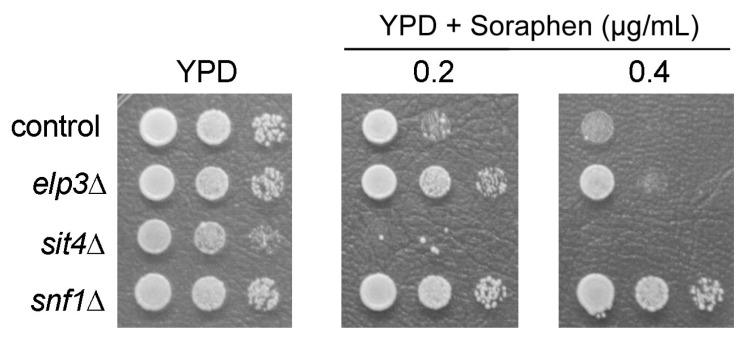
Soraphen A phenotype of *elp3* mutants. *Saccharomyces cerevisiae* strains BY4741 (control) and its isogenic *elp3*, *sit4* and *snf1* mutants were grown to stationary phase and seeded at a concentration of 10^7^, 10^6^ or 10^5^ cells/mL (from left to right) onto YPD medium plates containing the indicated soraphen A doses. Growth was recorded after three days. A representative result of three independent experiments is shown.

Since the tRNA overexpression results suggest that only part of the phenotypes of *sit4* mutants are caused by the defect in tRNA modification we further investigated a possible role of the modification in regulation of soraphen A resistance and LD dynamics, which are known to be strongly affected in *sit4* mutants [2]. We reasoned that a relevant contribution of the tRNA modification to these phenotypes should result in similar phenotypic changes in an *elp3* mutant. However, when we analyzed the soraphen A response of *elp3* mutants an opposite phenotype compared to that of *sit4* was detected (Figure 2).

**Figure 3:**
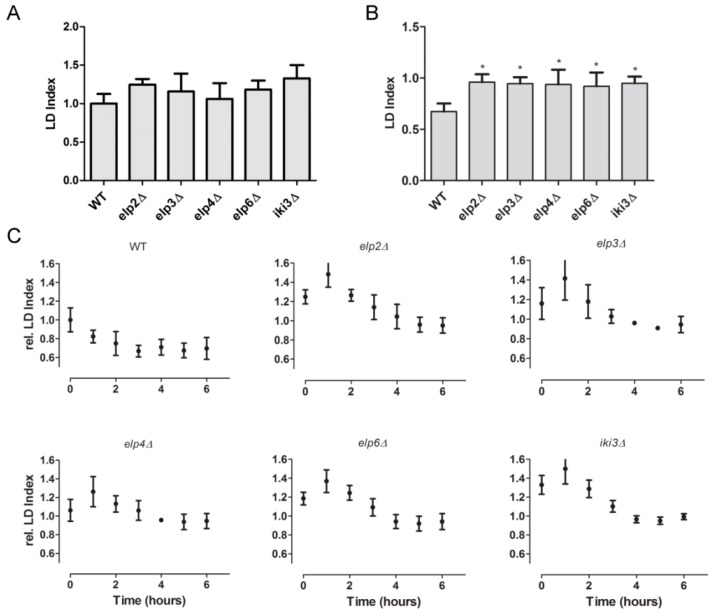
Lipid droplet (LD) content and dynamic change in Elongator mutants as compared to the wild type (WT). Elongator mutant strains were pre-grown to stationary phase (48 h) to attain a high LD content and then seeded at low density in YPD medium at 30 °C (**A**) Aliquots were withdrawn at stationary phase (48 h) or (**B**) after 5h of growth. (**C**) Lipid droplet dynamics of Elongator mutants was studied during the exponential phase (lipolytic phase) of growth. Stationary cells were seeded at 0.25 O.D 600 nm in YPD. Aliquots were withdrawn at the times indicated and fixed in formaldehyde 3.7%. Afterwards the LD index was measured with the liquid fluorescent recovery (LFR) assay using Bodipy 493/503 as a probe [2] and normalized to WT. The mean ± (standard deviation) S.D. of three independent experiments is shown (* *p* < 0.05).

This result and the absence of rescue effects by pQKE on the soraphen A trait of *sit4* (Figure 1C) suggest that this phenotype results from defects unrelated to the U_34_ modifications. However, the resistance of the *elp3* mutant also suggested a possible role of the modification in regulation of the soraphen A target Acc1 and potentially, in lipid biogenesis or dynamics in general. Soraphen A resistance of the *elp3* mutant was comparable to the *snf1* mutant (Figure 2), lacking negative regulation of Acc1 activity by phosphorylation [2], potentially indicating a relevant effect of the Elongator dependent tRNA modification on lipid metabolism. To further investigate whether Elongator dependent tRNA modification may influence dynamic changes in LD content, we analyzed LD levels in mutants lacking different Elongator subunits in comparison to the wild type. We scored lipolysis after re-inoculation of stationary phase cells, which maintain a high lipid droplet level, into fresh media over a time range of 6 h as previously described [32]. While wild type cells displayed a significant decrease in LD levels already in the first 2 h, all the analyzed Elongator mutants showed a significant delay in lipolysis (Figure 3).

It was shown previously that loss of the Snf1 kinase also results in higher than normal LD levels and soraphen A resistance, whereas *reg1*, *sap190* and *sit4* mutants exhibit lowered LD levels and soraphen A sensitivity [2]. Thus, in several mutants (*snf1*, *reg1*, *sap190*, *sit4*), changes in soraphen A resistance go hand-in-hand with reciprocal changes in LD content.

### 3.3. Screening Other tRNA Modification Mutants for Soraphen A Resistance

Since our data revealed an unexpected role of the Elongator dependent tRNA modifications in regulating LD dynamics and soraphen A resistance, we investigated whether other tRNA modification mutants might display abnormal LD levels, which were identified in a previous screening for changes in soraphen A resistance [33]. We assembled a set of mutants defective in most of the known tRNA modifications (excluding essential ones) and classified their soraphen A response. Based on this screening, we identified *deg1* and *tum1* as soraphen A resistant (Table 1). While Tum1 is (along with the Elongator complex) involved in synthesis of the mcm^5^s^2^U modification [22,34], Deg1 represents a well characterized pseudo-uridine synthase mediating the isomerization of uridines to pseudo-uridine (Ψ) in tRNA at positions 38 and 39 [23]. As reference for soraphen A sensitivity, we included *sit4* and *sap185* in this table.

### 3.4. Changes in Lipid Droplet Dynamics Upon Loss of Ψ38/39

**Figure 4:**
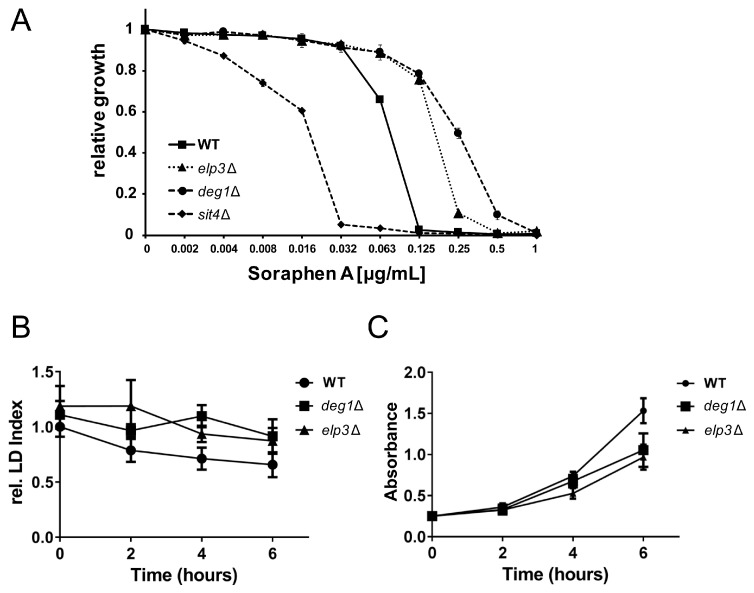
Soraphen A resistance and lipolysis defect in *deg1* mutants. (**A**) Microtiter based measurement of growth inhibition of indicated strains by soraphen A. Relative growth refers to optical density (O.D 600 nm) of microcultures with indicated concentrations of soraphen A as compared to soraphen A free cultures. Measurements were carried out in triplicate and shown as mean plus standard deviation. (**B**) Lipid droplet dynamics and (**C**) growth of BY4741 (WT) and its isogenic *elp3* and *deg1* mutants was determined during exponential phase of growth (6 h). The mean ± S.D. of three independent experiments is shown.

We confirmed the soraphen A resistance of the *deg1* mutant by using the liquid growth inhibition assay, which revealed a soraphen A resistance level slightly exceeding the one of *elp3* mutants (Figure 4A). Since a positive correlation between soraphen A resistance and LD content was observed in other mutants [2], we analyzed the LD dynamics in the *deg1* mutant in comparison to wild type and *elp3* cells. As shown in Figure 4B, *elp3* and *deg1* mutant cells indeed displayed a comparable defect in lipolysis upon re-inoculation from stationary phase to fresh media. Consistent with a slow growth phenotype of both mutants, a delay in resuming growth after refeeding in comparison to the wild type was observed by measuring the optical density concomitantly with LD quantification (Figure 4C).

### 3.5. Rescue of Lipolysis Defect of deg1 Mutants by tRNA Overexpression

**Figure 5:**
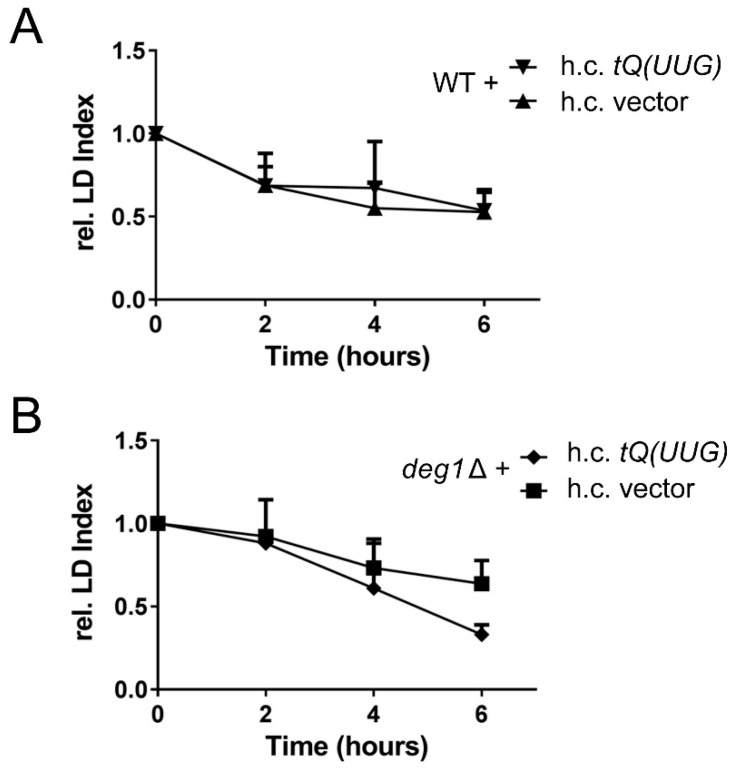
Suppression of lipolysis defects in *deg1* mutants by overexpression of tRNA^Gln^UUG. Wild type (WT) (**A**) and *deg1*∆ (**B**) cells were transformed with empty vector (h.c vector) or tRNA^Gln^UUG multicopy plasmid (h.c.*tQ(UUG)*). Strains were seeded at low density and the lipid droplet dynamics were assayed during exponential phase (6 h) in YPD medium. The mean ± S.D. of three independent experiments is shown.

The identification of two different tRNA modifiers (Elongator and Deg1), which impact on soraphen A resistance and LD levels in a similar manner, suggests that translational defects are responsible for the observed LD and soraphen A phenotypes. Translational defects of different tRNA modification mutants can be rescued by overexpression of those tRNAs that become functionally affected in the absence of the modifications [12,18]. For Deg1, previous work identified a single tRNA (tRNA^Gln^UUG) to be functionally impaired in the absence of the modification Ψ38/39 [25,35]. Hence, we analyzed whether lipolysis defects in *deg1* can be ameliorated upon overexpression of this tRNA. As shown in Figure 5, this is indeed the case. No significant difference in LD dynamics was observed in stationary phase wild type cells carrying empty vector or the tRNA^Gln^UUG overexpression construct upon refeeding (Figure 5A). In both, reduction of LD levels was observed. In contrast, *deg1* mutants carrying the tRNA overexpression construct display an increased reduction of LD levels at 4 h and 6 h after refeeding as compared to the empty vector control (Figure 5B). These results support the interpretation that tRNA modification defects influence LD dynamics by translational defects, since the lipolysis defect of *deg1* mutants can be ameliorated by overexpression of tRNA^Gln^UUG.

## 4. Discussion

Since deficiency of the protein phosphatase Sit4 induces multiple cellular effects, including the absence of mcm^5^s^2^U, mcm^5^U and ncm^5^U from tRNAs [22] which in itself already induces pleiotropic phenotypes [12,14,17], we aimed to dissect Sit4 functions relevant for regulating lipid metabolism and associated phenotypes. Our results demonstrate that Snf1 regulatory defects of *sit4* mutants [2] dominate over lipid specific effects induced by loss of tRNA modification alone. This conclusion is based on the comparison of soraphen A resistance and dynamic changes in lipid content in Elongator mutants lacking n/mcm^5^/s^2^U with previously described effects of the *sit4* mutation [2]. *sit4* mutants lacking n/mcm^5^/s^2^U modification in tandem with Snf1 overactivation display low LD levels and soraphen A sensitivity whereas single loss of the same tRNA modification in *elp3* mutants causes opposite effects (Figure 1 and Figure 3). Therefore, the former phenotypes appear to exclusively result from Snf1 overactivation in *sit4* without participation of the concomitant tRNA modification defect. In support of this notion, we also show that the soraphen A sensitivity of a *sit4* mutant cannot be rescued by tRNA overexpression, unlike other n/mcm^5^/s^2^U phenotypes (Figure 1) [12,17]. The same approach, however, also revealed a previously unknown soraphen A resistance and high lipid droplet phenotypes in Elongator mutants. The latter phenotype was specifically apparent when stationary phase cells were refed with fresh medium, which routinely induces fast mobilization of lipids from storage LDs [2,5]. Interestingly, a lipolysis defect was not only detected for mutants resulting in n/mcm^5^s^2^U deficiency but was also present in a *deg1* mutant lacking an entirely different tRNA modification (Ψ38/39). However, recent work identified mcm^5^s^2^U and Ψ38 to be particularly relevant for functioning of tRNA^Gln^UUG [18,25,35]. Since the lipolysis defect of *deg1* mutants was partially rescued by overexpression of tRNA^Gln^UUG, a specific translational defect of a Gln rich protein could be involved in the observed effects. Upon refeeding, triacylglycerol from lipid droplets is mobilized using Tgl3, Tgl4 and Tgl5 lipases [36]. Hence, a translation dependent defect in lipolysis at the level of refeeding could involve impaired expression of either of these proteins. However, none of these proteins are particularly Gln-rich (Tgl3 3.42% Gln, Tgl4 3.29% Gln, Tgl5, 4.53% Gln). Since the *deg1* mutation was shown to impair function of tRNA^Gln^UUG rather than tRNA^Gln^CUG [18,25,35], we also compared the relative usage of the two alternative Gln codons (CAA/CAG) in the *TGL3-5* genes. Relative CAA codon usage in *TGL3* (68.2%) and *TGL5* (67.6%) is close to the genome average (68.5%) and slightly increased in *TGL4* (73.3%). Hence, *TGL4* might be more sensitive to tRNA^Gln^UUG defects induced by *deg1* mutation compared to the other two genes. Alternatively, the delay of *elp3* and *deg1* mutants to resume exponential growth upon refeeding (Figure 4) may indicate a more global translational defect that causes a reduced ability to remodel-/reactivate gene expression and hence result in a delay of storage lipid consumption. Such delayed entry into exponential phase may contribute to an apparent lipolysis defect but is unlikely the reason for increased resistance against Acc1 inhibition observed in both *elp3* and *deg1* mutants (Figure 4A). In addition to defects in translation, loss of tRNA modification may also result in widespread transcriptional changes in a concerted cellular response against translational pausing and subsequently induced protein homeostasis defects [20,37,38,39]. Whether or not this includes transcriptional induction of genes relevant for lipogenesis and/or repression of genes required for lipolysis, remains to be determined. In general, a delay of storage lipid content reduction upon refeeding might also result from upregulated de novo lipid biosynthesis. If this would imply upregulation of Acc1 activity in tRNA modification mutants, soraphen A resistance could be expected. Of note, both *elp3* and *deg1* deletions were found to induce widespread metabolic changes [40,41], which may also include complex alterations ultimately leading to decreased lipolysis or increased lipogenesis as cells enter from stationary to new exponential growth phase. In addition, loss of the Tum1 enzyme required for thiolation of tRNA was recently shown to affect lipid metabolism [42], providing additional support for a general functional link between tRNA modification and lipid homeostasis.

## 5. Conclusions

The multifunctional protein phosphatase Sit4 is required for tRNA modification and modification independent regulatory processes in *S. cerevisiae*. Absence of Sit4 lowers cellular LD levels and results in increased sensitivity to acetyl-CoA carboxylase (Acc1) inhibition. It remained unknown whether these effects are due to loss of tRNA modification or due to modification independent functions of Sit4. We demonstrate in here that the effects on LD levels and susceptibility to Acc1 inhibition are caused by the tRNA modification independent effects of a *SIT4* gene deletion. However, absence of two distinct tRNA modifications results in defective lipolysis causing LD accumulation and resistance against Acc1 inhibition, which represent opposite effects of *sit4* deletion. Since the lipolysis defect in a tRNA modification mutant can be ameliorated upon overexpression of the tRNA affected by loss of the modification, translational inefficiency is assumed to induce these effects.

## Figures and Tables

**Table 1:** Soraphen A responses of tRNA modification and phosphatase mutants. Data were obtained from a soraphen A screening of a deletion library [33].

Gene	Nomenclature	Description	Sensitivity Score	Response
*DEG1*	*YFL001W*	depressed growth rate	+6	RESISTANT
*TUM1*	*YOR251C*	thiouridine modification	+2	RESISTANT
*SIT4*	*YDL047W*	suppressor initiation of transcription	−5	SENSITIVE
*SAP185*	*YKR028W*	Sit4 associated protein	−4	SENSITIVE
